# Large-conductance Ca^2 +^-activated K^+^ channel β1-subunit maintains the contractile phenotype of vascular smooth muscle cells

**DOI:** 10.3389/fcvm.2022.1062695

**Published:** 2022-12-09

**Authors:** Meili Wang, Shuanglei Li, Hongshan Liu, Mingyuan Liu, Jin Zhang, Yang Wu, Cangsong Xiao, Haixia Huang

**Affiliations:** ^1^Department of Physiology and Pathophysiology, School of Basic Medical Sciences, Capital Medical University, Beijing, China; ^2^Division of Adult Cardiac Surgery, Department of Cardiology, The Sixth Medical Center, Chinese PLA General Hospital, Beijing, China; ^3^Department of Vascular Surgery, Beijing Friendship Hospital, Capital Medical University, Beijing, China

**Keywords:** vascular smooth muscle cells, phenotype switching, BK_Ca_ channel, atherosclerosis, post-injury restenosis

## Abstract

**Background:**

Vascular smooth muscle cells (VSMCs) phenotype switching is very important during the pathogenesis and progression of vascular diseases. However, it is not well understood how normal VSMCs maintain the differentiated state. The large-conductance Ca^2+^-activated K^+^ (BK_Ca_) channels are widely expressed in VSMCs and regulate vascular tone. Nevertheless, there is limited understanding of the role of the BK_Ca_ channel in modulation of the VSMC phenotype.

**Methods and results:**

We assessed BK_Ca_ channel expression levels in normal and injured carotid arteries from rats of the balloon-injury model. A strong decrease of BK_Ca_-β1 was seen in the injured carotid arteries, accompanied by a parallel decrease of the VSMC contractile markers. BK_Ca_-β1 in primary rat aortic VSMCs was decreased with the increase of passage numbers and the stimulation of platelet-derived growth factor (PDGF)-BB. Conversely, transforming growth factor β upregulated BK_Ca_-β1. Meanwhile, the BK_Ca_-β1 level was positively associated with the levels of VSMC contractile proteins. Intravenous injection of PDGF-BB induced downregulation of BK_Ca_-β1 expression in the carotid arteries. Knockdown of BK_Ca_-β1 favored VSMC dedifferentiation, characterized by altered morphology, abnormal actin fiber organization, decreased contractile proteins expression and reduced contractile ability. Furthermore, the resultant VSMC dedifferentiated phenotype rendered increased proliferation, migration, enhanced inflammatory factors levels, and matrix metalloproteinases activity. Studies using primary cultured aortic VSMCs from human recapitulated key findings. Finally, protein level of BK_Ca_-β1 was reduced in human atherosclerotic arteries.

**Conclusion:**

BK_Ca_-β1 is important in the maintenance of the contractile phenotype of VSMCs. As a novel endogenous defender that prevents pathological VSMC phenotype switching, BK_Ca_-β1 may serve as a potential therapeutic target for treating vascular diseases including post-injury restenosis and atherosclerosis.

## Introduction

Vascular smooth muscle cells (VSMCs) retain plasticity of the arterial wall. Meanwhile, VSMCs can undergo reversible phenotype switching (1). Under physiological conditions, the vessels-resident VSMCs are differentiated, expressing a series of contractile proteins including smooth muscle myosin heavy chain, α-smooth muscle actin (α-SMA), smooth muscle 22α (SM22α), and calponin. However, when exposed to certain pathological factors, VSMCs can exhibit an adaptive reaction and lose the contractile phenotype. This process is called VSMC phenotype switching (2). The dedifferentiated phenotype is characterized by reduced expression of the VSMCs marker proteins. Concomitantly, dedifferentiated VSMCs show high proliferation rate, migration ability, enhanced synthesis of extracellular matrix and inflammatory factors, while they lose the regulating capability of vascular diameter and blood flow (3). Phenotypic modulation of VSMCs is an early event and critical pathological process of many cardiovascular diseases, including atherosclerosis (3), hypertension (2), aneurysm (4), and post-angioplasty restenosis (5). Thus, strategies blocking the VSMC phenotype switching may prevent the progression of these diseases (6–9). Various environmental stimuli, including mechanical injury, reactive oxidative species, growth factors/cytokines, and extracellular matrix instability, have been demonstrated to influence VSMC behavior and induce phenotype switching (2, 10, 11). Besides, it has been shown that transcriptional and epigenetic regulators can induce VSMC phenotypic transition (2). Nevertheless, how normal VSMCs maintain the contractile phenotype and confer vascular homeostasis remains largely unknown.

The differentiated VSMCs express a series of ion channels necessary for their unique contractile properties. VSMCs predominantly express large-conductance Ca^2+^-activated K^+^ (BK_Ca_) channels, which dampen depolarization-dependent activation of Ca^2+^ channels by hyperpolarizing membrane potential. Therefore, BK_Ca_ channels have a critical role in VSMC relaxation (12). In VSMCs, BK_Ca_ channel is a protein complex composed of two subunits, the pore-forming α-subunit (BK_Ca_-α) and the regulatory β1-subunit (BK_Ca_-β1) (13). We previously showed that BK_Ca_ channel activation exerts a beneficial effect in VSMC phenotypic transition by counteracting harmful stimulus (14). Moreover, studies have found that BK_Ca_ channel inhibition aggravates VSMC calcification (15). During calcification, VSMCs dedifferentiate to an osteochondrogenic phenotype. However, much more subtle phenotypic changes will also occur in VSMCs. Indeed, VSMC phenotype changes according to the local environmental factors as well as the different stages of development. In this study, we discovered that during the early stage of disease development, BK_Ca_-β1 negatively regulated VSMC dedifferentiation and maintained VSMCs in a quiescent contractile phenotype.

## Materials and methods

### Materials

An antibody against BK_Ca_-α (ab99046) used for immunohistochemical staining and western blot was purchased from abcam (Cambridge, UK). An antibody against BK_Ca_-β1 (APC-036) used for immunohistochemical staining and western blot was purchased from Alomone labs (Jerusalem, Israel). Antibodies against α-SMA (ab5694), SM22α (ab10135), and eIF5 (ab228874) used for western blot were purchased from abcam (Cambridge, UK).

### Animal experiments

All animal studies were approved by the Institutional Animal Care and Use Committee and Ethics Committee of Capital Medical University (Beijing, China) and were in strict accordance with the recommendation in the Guide for the Care and Use of Laboratory Animals of the National Institutes of Health. Healthy male Sprague-Dawley (SD) rats, aged 8–10 weeks (150–180 g), were obtained from the Animal Center of Capital Medical University and housed in an environment with standard conditions of humidity and room temperature and a light/dark cycle of 12/12 h.

### Balloon-injured rat model

The balloon-injured rat model was established as previously described (16). Briefly, male SD rats were anesthetized by intraperitoneal injection of pentobarbital sodium (40 mg/kg). A balloon catheter of 1.5-mm diameter (Medtronic, Minneapolis, MN, USA) was then inserted into the left external carotid artery lumen and advanced proximally to the common carotid artery lumen. The balloon was inflated to expand the carotid artery to 1.5 times its diameter and then gently pulled back with the rotational movement to the bifurcation. This pulling-back procedure was repeated twice. A similar operation that was performed on right carotid arteries, but without injury, served as a sham control. Two weeks later, the bilateral carotid arteries were harvested.

### Immunohistochemical staining

Tissues embedded in paraffin were cut into slices (7-μm in thickness) and subjected to immunohistochemical staining. Briefly, sections were incubated with corresponding antibodies at 4°C overnight and then HRP-labeled secondary antibodies at room temperature for 1 h. The nuclei were counterstained with hematoxylin.

### Immunofluorescence staining

For F-actin staining, VSMCs were fixed with 4% paraformaldehyde for 15 min and then permeabilized with 0.25% Triton X-100 in PBS for 5 min. Next, VSMCs were incubated with rhodamine phalloidin for 1 h at room temperature.

### Cell isolation and treatment

Primary rat VSMCs were isolated using the modified explant method (17). The thoracic aortas were used for the isolation. VSMCs were cultured in low-glucose Dulbecco’s Modified Eagle Medium (DMEM) supplemented with 10% fetal bovine serum (Gibco BRL, Grand Island, NY, USA) at 37°C in a humidified atmosphere containing 5% CO_2_. VSMCs passaged 3–6 were used for the experiments. To evaluate the effects of platelet-derived growth factor (PDGF)-BB or TGF-β, VSMCs at 80–90% confluence were subjected to serum starvation for 24 h followed by stimulation with the indicated concentration of PDGF-BB (25 μg/L) and TGF-β (2.5 μg/L) for 48 h.

### RNA interfering

Small interfering RNA- (siRNA-) targeting *KCNMB1* was designed and synthesized by OBiO (OBiO, China). Sequences corresponding to the small interfering RNA (siRNA) against *KCNMB1* were sense, 5′-CCUUGGUUGAUGUGAAGAATT-3′, and antisense, 5′-UUCUUCACAUCAACCAAGGTT-3′. siRNA transfection (50 nM) was performed using the Lipofectamine^®^3000 Transfection Reagent (Invitrogen, CA, USA), following the manufacturer’s instructions.

### Migration assay

A wound-healing assay was used for the assessment of VSMC migration. Briefly, VSMCs were seeded in a 6-cm culture dish. Then the confluent cell monolayers were scratched with a sterile 200 μL pipet tip. The cells were gently rinsed twice with PBS to remove floating cells and incubated with standard culture medium. The migration distance was monitored at 0, 6, 12, and 24 h of incubation. The longest distance of migration from the wound edge was measured (average of five independent microscope fields in each of the independent experiments).

### Western blot

Rat carotid tissue and VSMC extracts that contained equal amounts of total protein were separated by 12% SDS-PAGE gels and subsequently transferred onto PVDF membrane (Millipore, Billerica, MA, USA). The membranes were blocked with 5% milk (in TBST) for 1 h, followed by incubation with the corresponding primary antibodies at 4°C overnight. Following 1 h of incubation with HRP-conjugated secondary antibodies, the membranes were developed using an ECL reagent.

### Quantitative real-time PCR

Total RNA was extracted from cultured VSMCs with the TRIzol Reagent (Invitrogen, CA, USA). Equal amounts (1000 ng) were subjected to reverse transcription into cDNA using the ReverTra Ace^®^ qPCR RT kit (TOYOBO, Japan). Quantitation of all gene transcripts was done by qPCR using SYBR^®^ Green Realtime PCR Master Mix (TOYOBO, Japan) and ABI Step One Plus (Applied Biosystems, USA). The primer sequences used are as follows:

BK_Ca_-α: Forward: 5′-AGCGCGGTTAGTGGAAGAAA-3′
Reverse: 5′- ACTCTGGCAAGATCGTGTGG-3′
BK_Ca_-β1: Forward: 5′-CTATGGGCCCCAAATCCTCC-3′
Reverse: 5′-GAGCTGCCAAGACAGAGAGG-3′
PDGF-B: Forward: 5′-TCCGCTCCTTTGATGACCTT-3′
Reverse: 5′-TCCGACTCGACTCCAGAATGT-3′
TGF-β: Forward: 5′-ATACGCCTGAGTGGCTGTCTTT-3′
Reverse: 5′-AAAGCCCTGTATTCCGTCTCCT-3′
MCP-1: Forward: 5′-CAATGAGTCGGCTGGAGAAC-3′
Reverse: 5′-AGTGCTTGAGGTGGTTGTGG-3′
IL-6: Forward: 5′-GCTCTGGTCTTCTGGAGTTCC-3′
Reverse: 5′-GAGTTGGATGGTCTTGGTCCT-3′
IL-1α: Forward: 5′-ATCAGCACCTCACAGCTTCC-3′
Reverse: 5′-TCTCCTCCCGATGAGTAGGC-3′
TNF-α: Forward: 5′-CCAGGTTCTCTTCAAGGGACA-3′
Reverse: 5′-GTACTTGGGCAGGTTGACCTC-3′
GAPDH: Forward: 5′-TGTGAACGGATTTGGCCGTA-3′
Reverse: 5′-TGAACTTGCCGTGGGTAGAG-3′.

### Gelatin zymography

Vascular smooth muscle cells treated with BK_Ca_-β1 siRNA or scramble siRNA transfection were incubated in culture medium for additional 48 h. After that, the medium supernatant was separated by 10% SDS-PAGE gels containing 1 mg/ml gelatin (Sigma-Aldrich, MO, USA). The gel was washed with 2.5% Triton X-100 twice and then incubated in zymography buffer (50 mM Tris–HCl, 150 mM NaCl, 10 mM CaCl_2_, pH 7.5) for about 48 h. Following 6–8 h of incubation with Coomassie brilliant blue R250, the gels were imaged using the imaging instrument.

### Collagen gel contraction assay

Vascular smooth muscle cells were digested using trypsin and resuspended in culture medium at a density of 5 × 10^5^ cells/mL. Then the cells were mixed with 10xPBS, 0.1 M NaOH and type I collagen to prepare a collagen lattice. A total of 0.5 mL of the mixture were added to a 24-well plate and incubated for 1.5 h in the cell incubator. After that, each well was added with 0.5 mL of culture medium and the culture plate was incubated for another 24 h before taking pictures.

### Human samples

All human tissue samples were obtained from patients at the Chinese PLA General Hospital (Beijing, China). This study was conducted following the principles outlined in the Declaration of Helsinki and approved by the Medical Ethics Committee of Chinese PLA General Hospital (S2021-406-01). Informed consent was obtained from each participating patient. Internal mammary arteries (IMA) were obtained from patients undergoing coronary artery bypass surgery as control samples, and atherosclerotic arteries were collected from patients undergoing carotid endarterectomy (CEA).

### Isolation of primary vascular smooth muscle cells from human aortas

Primary cultures of aortic medial VSMCs were isolated from the thoracic aortas of human who underwent aortic valve replacement or aortic arch surgery using the modified explant method. The aortic specimen was rinsed in PBS to remove blood clots. After the adventitia with peripheral connective tissue was removed, the vessel wall was cut longitudinally and the intima was scraped gently. Then, the tunica media were cut into 1-mm^2^ explants and inoculated in a 25-cm^2^ flask. A total of 4–6 h later, culture medium was added carefully into the flask. The flask kept stationary in the incubator for 10 days until the cells grew around the explants. During this period, the culture media were refreshed every 3 days. The purity of VSMCs was tested by immunofluorescence staining for α-SMA and calponin. Isolated VSMCs were maintained in Kaighn’s Modification of Ham’s F-12 Medium (ATCC^®^ 30-2004™) and supplemented with 20% FBS (Gibco BRL, Grand Island, NY, USA) and 10% smooth muscle cell growth supplement (ScienCell).

### Statistical analysis

All the data were expressed as the means ± SEM. Protein band density was normalized to the corresponding control group and then to the mean of the corresponding control. Student’s *t*-test was used to analyze the differences between two groups. For a pairwise comparison of three or more groups, One-way ANOVA followed by the Student-Newman-Keuls test was used. The Chi-square test was used in the comparison of percentages between two groups. GraphPad Prism 9.0 (GraphPad Software, Inc., San Diego, CA, USA) was used for statistical analysis; *P-*value < 0.05 was considered statistically significant.

## Results

### BK_Ca_-β1 expression is decreased in the carotid arteries of balloon-injured rats

**FIGURE 1:**
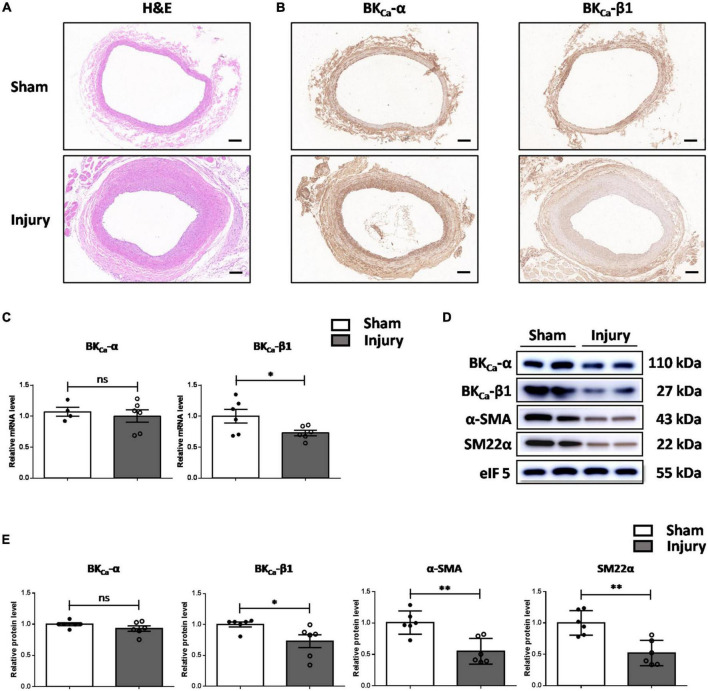
BK_Ca_-β1 expression is reduced in the carotid arteries of rat balloon-injured model. Representative images of H&E **(A)** and immunohistochemical staining of BK_Ca_−α and BK_ca_−β1 **(B)** in injured and sham-operated carotid arteries of rat balloon-injured model at 14 days post injury. Scale bar = 100 μm. **(C)** Quantified mRNA levels of BK_ca_−α and BK_ca_−β1 analyzed by qRT-PCR. Western blot images **(D)** and quantification **(E)** for indicated proteins. Eukaryotic initiation factor 5 (eIF5) was used as an internal control. Data are presented as means ± SEM (*n* = 4∼6 for each group, ns, not significant, *P < 0.05, **P < 0.01 vs. sham).

To assess the involvement of BK_Ca_-β1 in VSMC phenotype switching, we established a rat balloon-injury model. H&E staining showed that at 14 days post-injury, moderate to severe intimal hyperplasia developed in the injured carotid arteries (Figure 1A). No significant change was observed in the mRNA or protein level of BK_Ca_-α. However, the expression of BK_Ca_-β1 was downregulated, and the VSMC contractile proteins were reduced in injured vessels compared to sham-operated vessels (Figure 1). These results indicated that BK_Ca_-β1 downregulation was correlated with the changes of VSMC contractile phenotype markers (α-SMA and SM22α).

### BK_Ca_-β1 expression is regulated in the process of vascular smooth muscle cells phenotype switching *in vitro*

**FIGURE 2:**
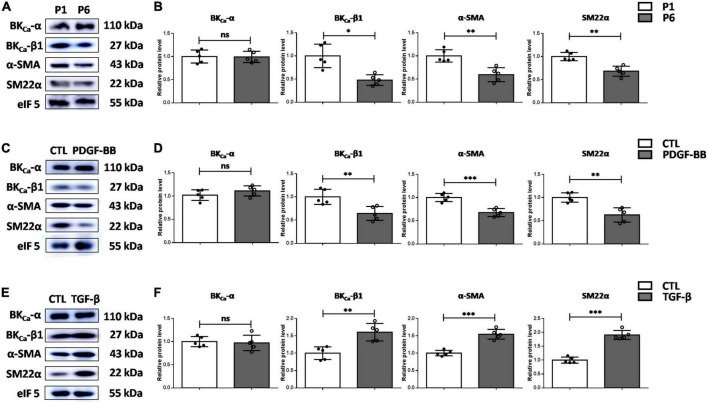
BK_Ca_-β1 expression is positively associated with VSMC differentiation markers *in vitro*. Western blot images **(A)** and quantification **(B)** of BK_ca_−α, BK_ca_−β1, and VSMC differentiation markers expression in VSMCs of passage 1 and passage 6. Western blot images **(C)** and quantification **(D)** of BK_ca_−α, BK_ca_−β1, and VSMC differentiation markers expression in VSMCs after PDGF-BB treatment (25 μg/L). Western blot images **(E)** and quantification **(F)** of BK_ca_−α, BK_ca_−β1, and VSMC differentiation markers expression in VSMCs treated with TGF-β (2.5 μg/L). VSMCs were subjected to serum-starvation for 24 h and then treated with the indicated stimulation for 48 h. Passages 3-5 of VSMCs were used in **(C)** through **(F)**. Eukaryotic initiation factor 5 (eIF5) was used as an internal control. Data are presented as means ± SEM (*n* = 5 for each group, ns, not significant, *P < 0.05, **P < 0.01, and ***P < 0.001 vs. control).

We then explored the association of BK_Ca_-β1 and the VSMC phenotype. As the cell passage increased, cultured primary rat VSMCs showed greatly reduced expression of differentiation markers, indicating the transformation from differentiated to dedifferentiated phenotype. In addition, compared with early passage VSMCs (passage 1, P1), late-passage VSMCs (passage 6, P6) showed markedly reduced expression of BK_Ca_-β1 (Figures 2A,B).

We then examined the response of BK_Ca_-β1 to vascular injury-associated stimuli. Plenty of studies have shown that PDGF-BB can stimulate proliferation and migration of VSMCs and induce the inhibition of VSMC markers expression (18, 19). Moreover, transforming growth factor β (TGF-β) is an effective VSMC differentiation factor *in vitro*, which transcriptionally regulates genes involved in proliferation and growth (20, 21). Our data indicated that BK_Ca_-β1 expression was reduced in cultured VSMCs due to PDGF-BB stimulation (Figures 2C,D). Inversely, TGF-β stimulation induced an opposite effect (Figures 2E,F). In addition, changes in BK_Ca_-β1 expression occurred in parallel with VSMC *trans-*differentiation states, as demonstrated by the changes in α-SMA and SM22α. Altogether, these data showed that BK_Ca_-β1 expression was closely related to the changes of VSMC contractile phenotype markers (α-SMA and SM22α).

### Elevated platelet-derived growth factor-BB mediated the downregulation of BK_Ca_-β1 expression *in vivo*

**FIGURE 3:**
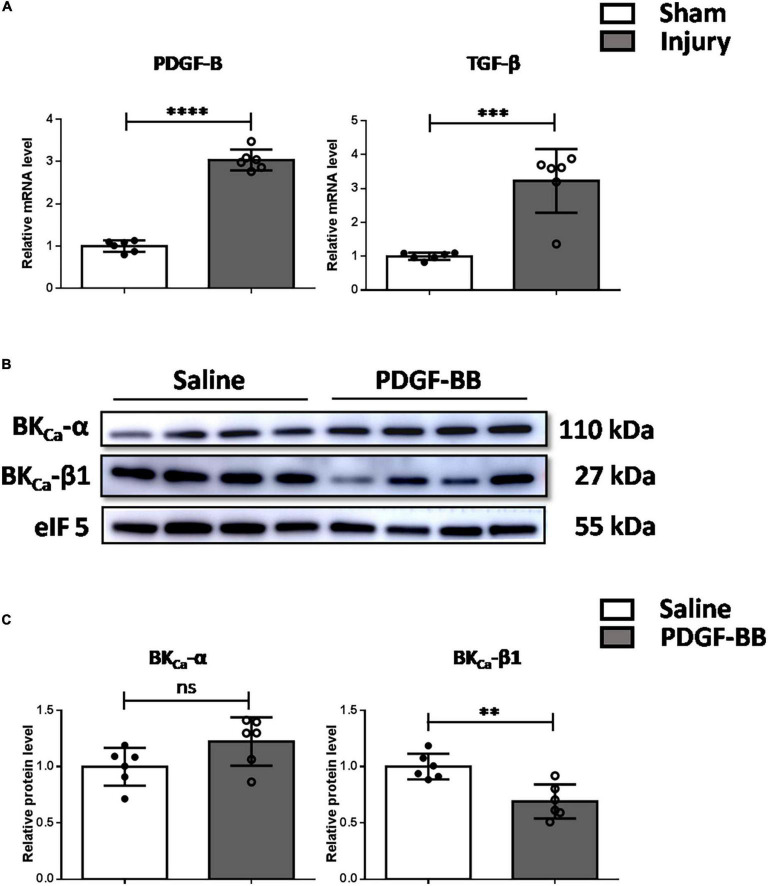
Decreased BK_ca_−β1 expression is mediated by elevated PDGF-BB *in vivo*. **(A)** Quantified mRNA levels of PDGF-B and TGF-β analyzed by qRT-PCR in injured and sham-operated carotid arteries. Western blot images **(B)** and quantification **(C)** of BK_ca_−α and BK_ca_−β1 in the carotid arteries of mice injected with saline or PDGF-BB *via* tail vein. Data are presented as means ± SEM (*n* = 6 for each group, ns, not significant, **P < 0.01, ***P < 0.001, and ****P < 0.0001 vs. control).

We further explored the mechanism of the downregulation of BK_Ca_-β1 expression *in vivo*. It has been reported that PDGF-BB and TGF-β both play important roles as endogenous growth regulatory factors during progressive intimal thickening after balloon angioplasty (22, 23). As Figure 3A shows, the expression of PDGF-B and TGF-β was both significantly increased after carotid injury. It is noteworthy that the change of PDGF-B is consistent with its effect on BK_Ca_-β1 expression *in vitro*. This result implied that PDGF-BB might be particularly important in BK_Ca_-β1 downregulation. Consistently, BK_Ca_-β1 expression was reduced in the carotid arteries of mice injected with PDGF-BB *via* tail vein (Figures 3B,C).

### BK_Ca_-β1 maintains the differentiated phenotype of vascular smooth muscle cells

**FIGURE 4:**
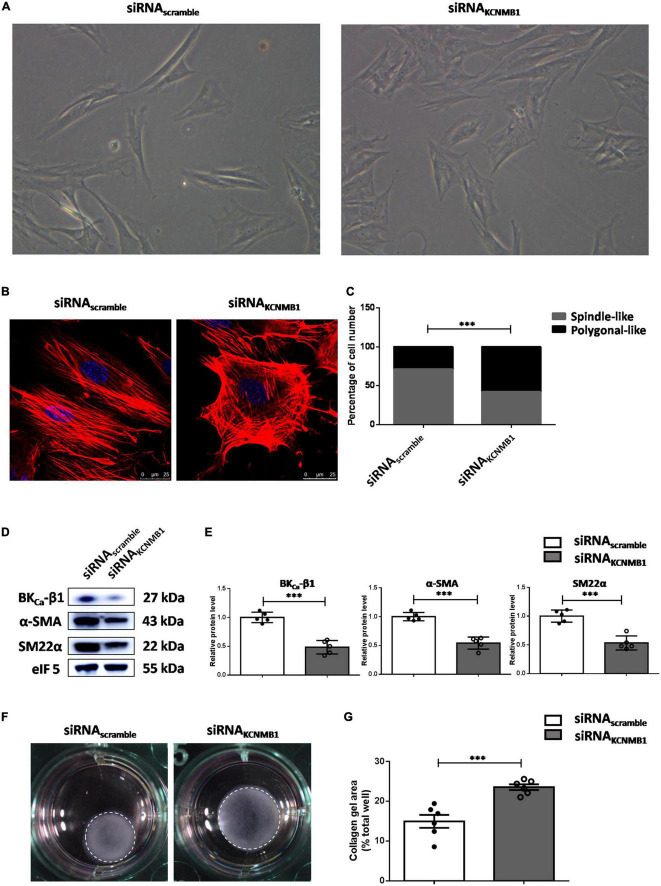
BK_Ca_-β1 is essential for VSMCs to maintain a differentiated phenotype. **(A)** Representative micrographs of VSMCs treated with BK_ca_−β1 siRNA or scramble siRNA transfection. **(B)** Representative immunofluorescent images of F-actin of VSMCs stained with phalloidin (red). Nuclei were stained with DAPI (blue). Scale bar = 25 μm. **(C)** The percentage of spindle-like or polygonal-like cells in each group. Western blot images **(D)** and quantification **(E)** of BK_ca_−β1, α-SMA, and SM22α. Representative images **(F)** and quantification **(G)** of the areas of collagen gel in each group. The size of the collagen gel in each well was calculated compared to the total area of the well. Data are presented as means ± SEM (*n* = 5∼6 for each group, ***P < 0.001 vs. scramble).

To obtain direct evidence supporting the role of BK_Ca_-β1 in VSMC phenotype switching, we used siRNA for the BK_Ca_-β1 (siRNA*_*KCNMB1*_*). Morphologically, scramble siRNA-treated VSMCs were spindle-like, while BK_Ca_-β1-targeting siRNA-treated VSMCs were polygonal (Figure 4A). Dedifferentiated VSMCs show impaired actin filament formation (24). Phalloidin staining showed that BK_Ca_-β1 knockdown disintegrated actin fibers into short and disorganized fibers, resulting in polygonal-shaped VSMCs (Figures 4B,C). Besides, the protein levels of α-SMA and SM22α decreased after siRNA treatment (Figures 4D,E), indicating that BK_Ca_-β1 was required for differentiation markers expression. To assess the contractile function of the VSMCs, the collagen gel contraction assay was performed. In accordance, VSMCs with BK_Ca_-β1 silencing exhibited reduced contractility (Figures 4F,G). These data suggested that BK_Ca_-β1 was necessary for the maintenance of the VSMC differentiated phenotype.

### BK_Ca_-β1 knockdown facilitates dedifferentiated phenotype of vascular smooth muscle cells

**FIGURE 5:**
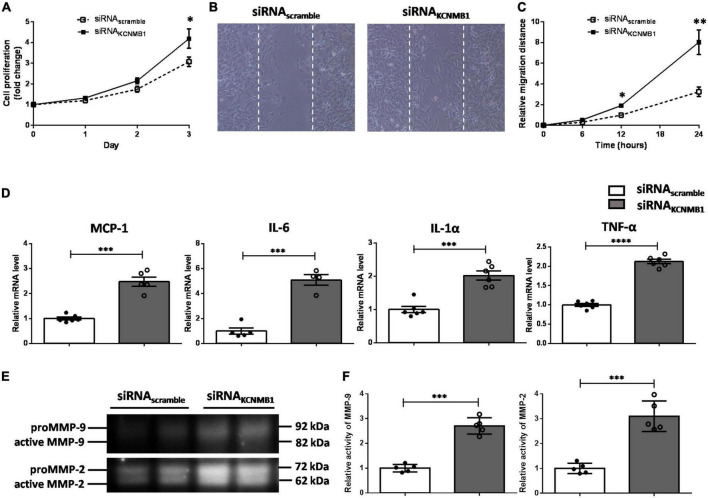
BK_Ca_-β1 knockdown induces proliferative, migratory, and synthetic phenotype of VSMCs. **(A)** Proliferation curves of VSMCs transfected with BK_ca_−β1 or scrambled siRNA. Representative images **(B)** and quantification **(C)** of migration assay. **(D)** Quantified mRNA levels of inflammatory factors analyzed by qRT-PCR. Representative images **(E)** and quantification **(F)** of activities of MMP-9 and MMP-2 tested using gelatin zymography. Data are presented as means ± SEM (*n* = 5∼6 for each group, *P < 0.05, **P < 0.01, ***P < 0.001, and ****P < 0.0001 vs. scramble).

Dedifferentiated VSMCs display an enhanced proliferative and migratory capacity. As shown in Figure 5A, BK_Ca_-β1 silencing VSMCs showed an increased capacity to proliferate. Scratch-wound assays showed higher migration ability of VSMCs with BK_Ca_-β1 knockdown compared to control cells (Figures 5B,C). Another hallmark of the dedifferentiated VSMCs is the enhanced proinflammatory response and greater proteolytic activity. As Figure 5D shows, the mRNA levels of MCP-1, IL-6, IL-1α, and TNF-α were markedly increased after BK_Ca_-β1 siRNA treatment. In addition, increased activity of matrix metalloproteinases (MMPs, including MMP-9, and MMP-2) was observed using the method of gelatin zymography (Figures 5E,F). Collectively, the dedifferentiated phenotype secondary to BK_Ca_-β1 knockdown exhibited an enhanced proliferative, migratory and synthetic response.

### BK_Ca_-β1 reduction correlates with human vascular smooth muscle cell dedifferentiation and atherosclerosis

**FIGURE 6:**
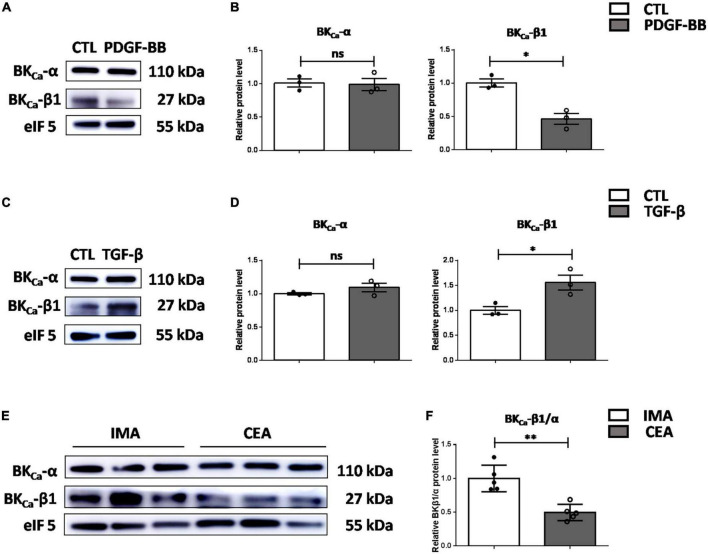
Decreased BK_ca_−β1 expression is correlated with VSMC dedifferentiation and atherosclerosis in human. Western blot images **(A)** and quantification **(B)** of BK_ca_−α and BK_ca_−β1 expression in human primary aortic VSMCs after PDGF-BB treatment (25 μg/L). Western blot images **(C)** and quantification **(D)** of BK_ca_−α and BK_ca_−β1 expression in human primary aortic VSMCs treated with TGF-β (2.5 μg/L). VSMCs were subjected to serum-starvation for 24 h and then treated with the indicated stimulation for 48 h. Passages 3–5 of VSMCs were used. **(E)** Representative western blot images of the protein levels of BK_ca_−α and BK_ca_−β1 in lysates of human carotid endarterectomy artery (CEA) and control internal mammary arteries (IMA). **(F)** Quantification was performed by calculating the ratio between the levels of BK_ca_−β1 and BK_ca_−α. Eukaryotic initiation factor 5 (eIF5) was used as an internal control. Data are presented as means ± SEM (n = 3∼5 for each group, ns, not significant, *P < 0.05, **P < 0.01 vs. control).

To verify the role of BK_Ca_-β1 in the VSMC identity in human, we isolated and cultured primary aortic VSMCs from patients who underwent aortic valve replacement or aortic arch surgery (Supplementary Figure 1). As the results showed, PDGF-BB markedly decreased, whereas TGF-β stimulation significantly increased BK_Ca_-β1 expression, which paralleled the VSMC *trans-*differentiation state (Figures 6A–D). We further collected atherosclerotic tissue samples from human. Clinical information of human subjects recruited for this study is shown in Table 1. Notably, protein level of BK_Ca_-β1 was significantly reduced in the arteries of patients who underwent CEA compared with control IMA used for coronary artery bypass graft surgery (Figures 6E,F). Together, these results indicated that reduced BK_Ca_-β1 expression was correlated with VSMC dedifferentiation in human.

**TABLE 1:** Clinical characteristics of the patients.

Sample characteristics	IMA (*N* = 5)	CEA (*N* = 5)
Gender (male)	4 (80)	5 (100)
Age	69.60 ± 3.203	66.80 ± 4.042
History of hypertension	3 (60)	5 (100)
History of diabetes	2 (40)	1 (20)
History of smoking	3 (60)	3 (60)
History of drinking	1 (20)	2 (40)
Glucose, mmol/L	6.394 ± 0.8446	7.920 ± 1.883
TC, mmol/L	3.556 ± 0.2765	4.720 ± 0.4893
TG, mmol/L	1.558 ± 0.1492	1.340 ± 0.3140
HDL, mmol/L	1.010 ± 0.1220	1.100 ± 0.1581
LDL, mmol/L	1.894 ± 0.2640	2.880 ± 0.3277*
Scr, μmol/L	85.72 ± 11.38	80.86 ± 6.464
BUN, mmol/L	6.452 ± 0.6659	5.460 ± 0.8773

IMA, internal mammary artery; CEA, arteries undergoing carotid endarterectomy; TC, total cholesterol; TG, triglyceride; HDL, high density lipoprotein; LDL, low density lipoprotein; Scr, serum creatinine; BUN, blood urea nitrogen. *P < 0.05.

## Discussion

Differentiated VSMCs share definite contractile characteristics, whereas dedifferentiated VSMCs can show different functional states or phenotypes. According to the functional characteristics, these VSMCs can be osteochondrogenic, proliferative, migratory, synthetic, and inflammatory (2). Although not depicted, there may be distinct modulation for VSMC phenotypes with changing environmental cues. In the early stages of vasculopathy, VSMCs proliferate rapidly and show enhanced migratory capacity. As the disease progresses, VSMCs synthesize a large number of hazardous substances including extracellular matrix protein, inflammatory factors and MMPs. It is generally believed that BK_Ca_ channels play a central role in controlling smooth muscle cell contraction. Recent studies have indicated that BK_Ca_ channel activation ameliorates VSMCs calcification, functionally related to the α-subunit (15). It is worth noting that during calcification, VSMCs transform into osteoblast/chondrocyte-like cells. However, the possible involvement of the BK_Ca_ channel in the VSMC phenotype switching toward other pathological phenotypes has not yet been explored.

Restenosis often occurs after by-pass surgery, angioplasty interventions and vascular injury, during which VSMCs are characterized by pathological proliferation and migration (25). During restenosis, VSMCs can undergo a transient modification of their phenotype, which is originally prepared to repair the vascular injury. The rat model of vascular injury is often used to describe the initial VSMC phenotypic modulation associated with neointimal hyperplasia and can closely mimic restenosis in humans. With the use of the balloon catheter injury model, it has been demonstrated that neointimal formation after angioplasty is associated with downregulated BK_Ca_ expression (26). During this process, the mature contractile VSMCs transform into proliferating neointimal VSMCs. Herein, we further proved that the decreased expression of BK_Ca_-β1 was earlier and more obvious (Figure 1). At the same time, reduced expression of VSMCs contractile proteins was observed. During neointima formation, complex interactions between growth-stimulating molecules can promote the proliferation and migration of VSMCs (27). Therefore, reduced BK_Ca_-β1 expression in the neointima might be mediated by many mitotic factors. As reported, TGF-β plays different roles *in vitro* and *in vivo*. *In vitro*, TGF-β can promote SMCs to transform into contractile phenotype and inhibit proliferation (24). Nevertheless, *in vivo*, TGF-β can promote the formation of neointima and aggravate the restenosis of blood vessels. Besides, purified, recombinant TGF-β stimulated neointimal, but not medial, SMC proliferation *in vivo* (22). This suggests that functional differences between neointimal and medial SMCs may extend to the level of growth control. In addition, TGF-β can stimulate or inhibit VSMC proliferation depending on cell density (28–32). All these discrepancies above suggest that the phenotype and biological behavior of SMCs are affected by the surrounding microenvironment and there are complex regulations in the body. Anyhow, our results indicated that BK_Ca_-β1 is consistently and positively associated with the contractile phenotype of VSMCs *in vitro* and *in vivo*.

Numerous studies have confirmed that PDGF-BB can significantly promote the proliferation and migration of VSMCs after arterial injury (18, 33). *In vitro*, PDGF-BB stimulation significantly reduced the expression of SM α-actin, SM MHC, and SM22α in the cultured VSMCs (34–37). Our research also proves this (Figures 2C,D). Research has demonstrated that PDGF-BB suppresses VSMC contractile genes expression through the ERK1/2-MAPK pathway, which leads to the dissociation and nucleation of myocardin and SRF. Consistently, previous report has shown that BK_Ca_-β1 expression was also driven by myocardin and SRF (38). Thus, we speculated that the ERK1/2-MAPK pathway would also mediate the downregulation of BK_Ca_-β1 expression. Whether other mechanisms are involved needs further exploration.

Decreased expression or increased degradation of BK_Ca_-β1 has been related to increased vascular tension and hypertension (39). BK_Ca_-β1 expression is decreased in VSMCs from hypertensive (39, 40), aging (41), diabetic (42), and hypoxic rodent models (43). It has also been shown that vascular BK_Ca_ channel function is impaired in Type 1 diabetic mice and Type 2 diabetic patients, mainly due to the marked decrease of BK_Ca_-β1 (44, 45). As direct causal evidence, BK_Ca_-β1 deletion in mice leads to increased arterial tension and elevated blood pressure (46, 47). In high-fat-diet mice, BK_Ca_-β1 deficiency exacerbates vascular remodeling and fibrosis (48). Most importantly, loss-of-function mutations in the *KCNMB1* lead to hypertension and renal diseases in humans (49). Inversely, a gain-of-function mutation in the *KCNMB1* (E65K) is associated with low incidence of diastolic hypertension (50–52). All of the above findings suggest that decreased BK_Ca_-β1 expression can promote the occurrence and development of vascular diseases. To date, there are no reports on the role of BK_Ca_-β1 in the modulation of the VSMC phenotype. Herein, for the first time, we discovered that BK_Ca_-β1 was significantly correlated with the phenotype switching of VSMCs. In addition, we first provided direct evidence that BK_Ca_-β1 knockdown can drive VSMC dedifferentiation. More importantly, we confirmed that decreased BK_Ca_-β1 expression was closely related to the dedifferentiation of VSMCs and atherosclerosis in human. Yet, the exact mechanisms underlying BK_Ca_-β1 deficiency-induced phenotype switching remain to be studied.

BK_Ca_ channel is composed of BK_Ca_-α and BK_Ca_-β. In the vascular system, BK_Ca_-β1, encoded by *KCNMB1*, is the major subtype in VSMCs. BK_Ca_-α forms a functional structure and displays the essential properties of native BK_Ca_ channels: voltage and Ca^2+^ sensitivity, K^+^ selectivity and large conductance (53). As for B K_Ca_-β1, it confers the BK_Ca_ channel with higher Ca^2+^ and voltage sensitivity, making this channel more efficient in VSMC functions (54). Moreover, BK_Ca_-β1 regulates BK_Ca_-α expression on the membrane *via* regulating endocytic trafficking signaling (55). Our previous studies identified the mechanosensitivity of the STREX-lacking BK_Ca_ channel in the colonic smooth muscle (56) and further verified that BK_Ca_-β1 is involved in the regulation (13). Studies have revealed that BK_Ca_ channel activity is significantly affected by the expression of BK_Ca_-β1, and changes in cellular function impaired by BK_Ca_-β1 seem to be more significant than the functional differences among variations in BK_Ca_-α. In accordance with this, many hormones and curative medicines are found to enhance the BK_Ca_ channel activity by acting on or interacting with BK_Ca_-β1, and BK_Ca_-β1 targeted compounds prove to be more favorable for VSMCs disorders where its expression is restricted (57). Notably, our present study showed that BK_Ca_-β1 deficiency is specifically involved in the dedifferentiation of VSMCs. In addition, downregulation of BK_Ca_-β1 was characteristic for proliferative and migratory phenotypes of VSMCs.

Taken together, this study demonstrated that BK_Ca_-β1 is an important regulator of VSMC identity by preventing its phenotype switching. These findings reveal a self-protective mechanism of VSMCs against harmful environmental stimuli and support a protective role of BK_Ca_ channel activation in various vascular diseases in humans. Targeting BK_Ca_-β1 to precisely modulate BK_Ca_ activity may provide novel therapeutic strategy for post-injury restenosis and atherosclerosis.

## Data availability statement

The raw data supporting the conclusions of this article will be made available by the authors, without undue reservation.

## Ethics statement

The studies involving human participants were reviewed and approved by the Medical Ethics Committee of Chinese PLA General Hospital. The patients/participants provided their written informed consent to participate in this study. The animal study was reviewed and approved by the Institutional Animal Care and Use Committee and Ethics Committee of Capital Medical University (Beijing, China). Written informed consent was obtained from the individual(s) for the publication of any potentially identifiable images or data included in this article.

## Author contributions

MW, SL, CX, and HH designed the study. MW and SL performed the experiments and analyzed the data. HL, ML, JZ, and YW analyzed the data. MW wrote the manuscript. HH finalized the manuscript. All authors critically revised the manuscript and approved the submitted version.
