# Supplementary material for: Large-conductance Ca2 +-activated K+ channel β1-subunit maintains the contractile phenotype of vascular smooth muscle cells
Source: Front Cardiovasc Med. 2022 Dec 9;9:1062695. doi: 10.3389/fcvm.2022.1062695 (PMC9780463; doi:10.3389/fcvm.2022.1062695)
Supplement: Supplementary file 1 [file Data_Sheet_1.docx]

**
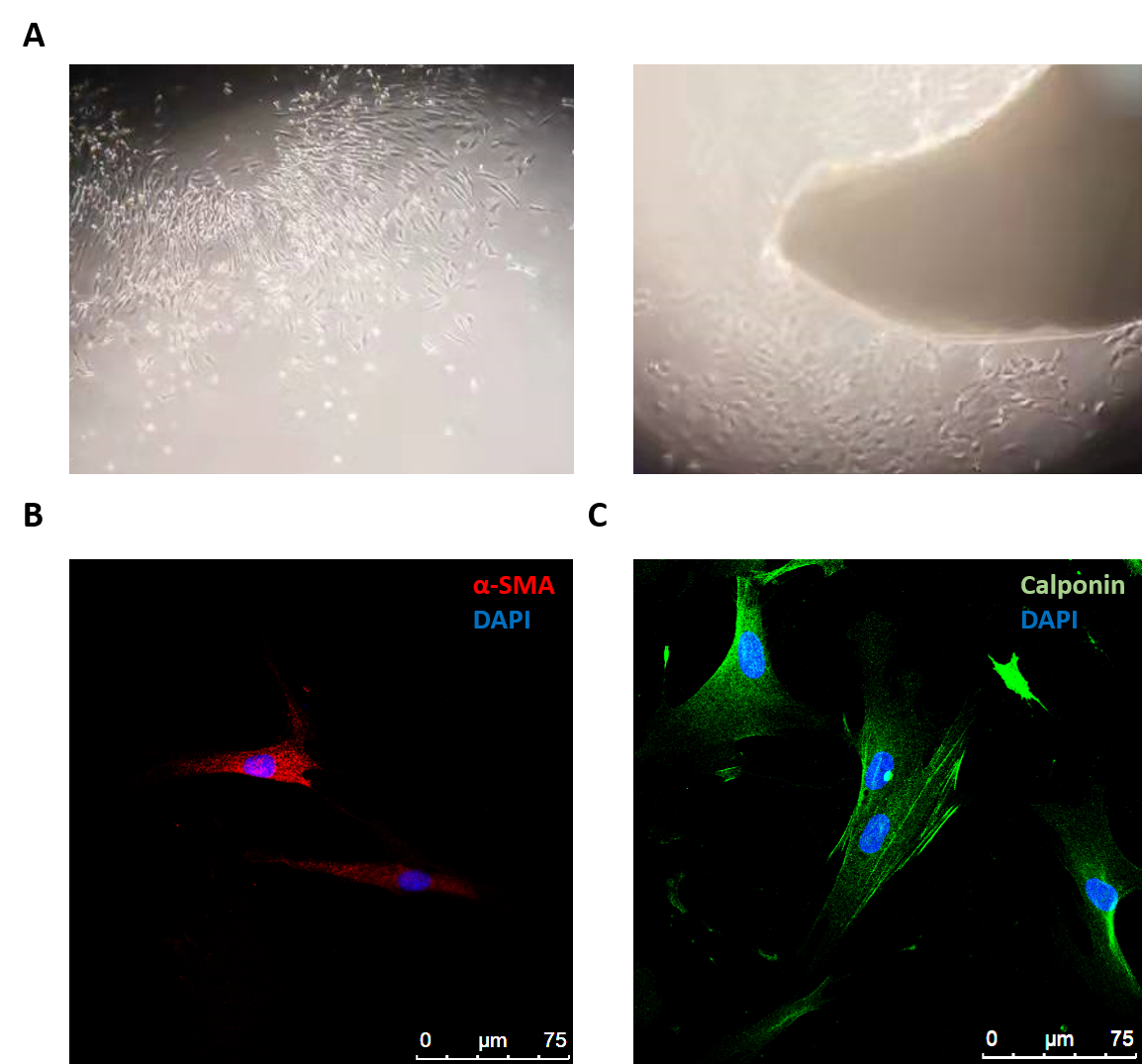
**

**Figure S1. Isolation and identification of primary VSMCs from human aortas.**

**A.** Light microscope images of cultured smooth muscle cells from human aortic tissues. The immunofluorescent staining of α-SMA **(B, red)** and calponin **(C, green)** in VSMCs. Nuclei were counterstained with DAPI (blue).
